# Interpretation services used for non-English language speaking patients with head and neck cancer: Findings from a tertiary London cancer centre

**DOI:** 10.1016/j.oor.2024.100229

**Published:** 2024-03

**Authors:** Maesaya Chartkul, Florence Cook, Roganie Govender

**Affiliations:** a Division of Medicine, University College London, Gower Street, WC1E 6BT, UK; b Head and Neck Unit, University College London Hospital NHS Foundation Trust, 250 Euston Road, London, NW1 2PG, UK; c Head & Neck Academic Centre, Division of Surgery and Interventional Science, University College London, Charles Bell House, 43-47 Foley Street, W1W 7TS, UK

**Keywords:** Language interpreter practices, Interpretation services, Communication barriers, Health services accessibility, Translating, Language

## Abstract

**Background:**

Treatment for head and neck cancer (HNC) can lead to profound changes in swallowing function and nutrition. UK HNC standards recommend informational counselling is provided pre-treatment by the dietitian and speech and language therapist (SLT). Non-english speaking patients treated in English speaking countries have poorer treatment outcomes. UK guidance recommends all patients should have access to interpretation services where required. This audit aimed to investigate the language needs and utilisation of interpretation services for patients with HNC attending pre-treatment clinics.

**Methods:**

A retrospective casenote audit over two-years (2021–2023) of all patients attending pre-treatment clinics in a centralised London cancer centre. Information was collected from electronic hospital records on demographics, disease/treatment characteristics, first language; categorised as English primary language (EPL), non-English primary language (NEPL) and interpretation requirements; formal (professional interpreters) or informal (patients friends/relatives). Data was processed and collated using Microsoft Excel (Version 2308). Descriptive statistics were conducted using Excel Analysis ToolPak.

**Results:**

408 patients were included. Most patients were male (67%), aged 61 ± 13 years. 18% (n = 74) had NEPL; 58 required interpretation services. Formal interpretation service was provided in 43% (n = 25), informal services in 33% (n = 19), and no service in 24% (n = 14). Non-adherence to formal services included lack of/inaccurate documentation of language needs, patient preference and lack of available interpreters.

**Conclusion:**

This audit indicated that barriers exist in utilising formal interpretation services. Future research should investigate how this can be optimised to ensure necessary language support is provided consistent with guidelines that promote equality, diversity, and inclusion.

## Highlights

• Non-English speaking patients treated in English speaking countries have poorer treatment outcomes and satisfaction of utilising healthcare services.
• United Kingdom national guidance recommends all patients have access to interpretation services where required.
• Head and neck cancer (HNC) profoundly impacts everyday functions including eating, drinking and communication.
• At a large, metropolitan hospital in London, 14% of patients with HNC had language barriers, and of these, 43% received formal interpretation.
• Reasons included lack of available interpreter, poor documentation of language requirements in hospital records and patient preference for non-formal services.

## 1 Introduction

Head and neck cancer (HNC) is the eighth most common cancer in the UK, with 12,400 patients diagnosed annually [1]. Treatment includes surgery and/or radiotherapy (with or without chemotherapy) which can result in profound changes in function including swallowing, speech, and appearance [2,3].

Patients with HNC face unique nutritional challenges due to the impact of the disease itself and subsequent treatment on eating and drinking [4]. This can lead to the development of malnutrition; this affects approximately 60% of patients with HNC at diagnosis and is directly associated with treatment outcomes, quality of life and survival [5,6]. Nutrition support is often required at any stage of the care pathway to supplement nutritional needs. This includes enteral tube feeding which may be required long-term, and in some cases, a life-long period due to impact of treatment on swallowing function [4].

National HNC standards recommend that patients undergoing treatments that will likely impact on nutrition and swallowing should be offered a pre-treatment appointment with the dietitian and speech and language therapist (SLT). The purpose of this appointment is to conduct a baseline nutrition and swallowing assessment alongside informational counselling on the impact of planned treatment on speech and swallowing. This includes anticipated requirements for tube feeding on a short- and long-term basis. At our centre, this appointment is conducted jointly by the dietitian and SLT.

Existing research indicates that non-English speakers are less likely to follow multidisciplinary team treatment recommendations [7] resulting in poorer outcomes [8]. National Institute for Health and Care Excellence (NICE) guidelines [CG138] for improving the experience of care for people using adult NHS services' state that healthcare providers have a responsibility to ensure all patients have access to interpretation services when this is required [9]. This aligns with NHS England guidance for commissioners on interpreting and translation which states that *‘whilst not being able to speak English is not a protected characteristic; defined under the Equality Act 2010, section 13G of the NHS Act 2006 states there must be regard to reducing inequalities between patients with respect to their ability to access health services and interpretation and translation should be provided free at point of delivery, be of high quality, accessible and responsive to a patient's linguistic needs'* [10].

Limited literature exists that determines the interpretation requirements of patients with HNC. Given the complexity and impact of treatment for HNC, it is imperative that patients understand information provided as part of the informed consent process. Consequently, we sought to address this gap by auditing the utilisation of interpretation services in the pre-treatment clinic within a large metropolitan hospital and centralised London cancer centre serving a diverse population. This audit had three objectives.

1 To identify the interpretation needs of patients attending the pre-treatment clinic.
2 To determine whether patients who required interpretation services received them in accordance with NICE guideline recommendations.
3 To identify reasons for the absence of interpretation services, where these were indicated but not forthcoming.

## 2 Materials and methods

This clinical audit was registered in the hospitals Clinical Support Services Division and ethical approval was not required. Patients were included if they were adults (≥18 years) with a diagnosis of HNC and scheduled for joint dietetic and SLT pre-treatment clinics. Patients were excluded if they did not attend the appointment or attended with an unknown diagnosis and/or treatment plan.

Data was collected retrospectively using electronic patient records over two years (January 1st, 2021, to January 31st, 2023). Demographic data was collected on age, sex, ethnicity, occupation and social circumstances. Information was also gathered on primary diagnosis, treatment characteristics, nutritional status, first language, and interpretation requirements. Interpretation services provided were categorised as formal (professional interpreters booked from the hospitals recommended language line services) or informal (using patients friends/relatives as translators). Language was categorised as English primary language (EPL), non-English primary language (NEPL) (languages other than English as their first language). In NEPL, patients with English language preference were defined as those as not requiring an interpreter, while patients with non-English language preference were defined as those requiring an interpreter in the pre-treatment proforma [[11], [12], [13]]. Nutritional status was defined according to criteria in the NICE (2006) Nutrition support for adult's guideline [CG32] [14] with malnutrition defined as those with a Body Mass Index (BMI) of <18.5 kg/m [2] *or* unintentional weight loss >10% within the last 3–6 months *or* BMI<20 kg/m [2] and unintentional weight loss >5% within the last 3–6 months. Those at risk of malnutrition were defined as having eaten little/nothing for >5 days and/or likely to eat little/nothing for the next 5 days or longer *or* having poor absorptive capacity, and/or have high nutrient losses and/or have increased nutritional needs from causes such as catabolism.

Data were processed, collated, and analysed using Microsoft® Excel® for Microsoft 365 MSO (Version 2308). Descriptive statistics were conducted using Microsoft Excel Analysis ToolPak.

## 3 Results

Over the two-year period (January 2021 to January 2023) 465 patients were identified with 408 patients included in the audit. Thirty-nine patients were removed before screening, in accordance with NHS data opt-out for reviewing records. A further 18 patients were excluded due to not meeting the inclusion criteria (n = 3 <18 years; n = 13 non-attendance and n = 2 unknown treatment plan). In the 408 cases, there were 444 visits, as some patients had a second pre-treatment visit for adjuvant treatment e.g. post-operative radiotherapy. The following results relate to the first visit of the 408 patients.

Table 1 depicts demographic data. Most patients were male (67%), and the mean age was 61 ± 13 years. EPL accounted for the majority (n = 334, 82%), while the remaining were NEPL (n = 74, 18%). Mean age of NEPL was (58 ± 13 years), lower than EPL (61 ± 12 years, p < 0.05). Professional occupations were more common (23%) in the EPL group compared to the and NEPL (12%) (p < 0.05). Regarding living arrangements, most patients lived with family (74%), with NEPL showing a higher percentage of living with family (86%) than EPL (71%, p < 0.05). Surgery was the most common treatment modality (41%), followed by radiotherapy or chemoradiotherapy (32%). Surgical treatment was more common in NEPL (55%) than EPL (38%, p < 0.05) and lower anticipated need for a gastrostomy tube (20%) compared to EPL (34%, p < 0.05).

**Table 1:** Patient demographic data from January 2021 to January 2023 including patients who primary language was, and was not English.

	Total (n = 408)	EPL (n = 334)	NEPL (n = 74)	p-value
**Age (average)**				
Year, mean (SD)	61 (13)	61 (12)	58 (13)	<0.05
**Sex, n (%)**				
Male	275 (67)	235 (70)	40 (54)	<0.05
Female	132 (32)	99 (30)	34 (46)	
**Occupation, n (%)**				
Non-Professional	43 (11)	37 (11)	6 (8)	0.45
Professional	92 (23)	83 (25)	9 (12)	<0.05
Retiree	50 (12)	47 (14)	3 (4)	<0.05
Unemployed or student	16 (4)	11 (3)	5 (7)	0.16
Not recorded	207 (51)	156 (47)	51 (69)	<0.05
**Relationship status, n (%)**				
Married or cohabiting	216 (53)	182 (54)	34 (46)	0.18
Prefer not to say	43 (11)	31 (9)	12 (16)	0.08
Single or never married	16 (4)	15 (5)	1 (1)	0.21
Divorced or separated	11 (3)	10 (3)	1 (1)	0.43
Widowed	11 (3)	10 (3)	1 (1)	0.43
Not recorded	111 (27)	86 (26)	25 (34)	0.16
**Living arrangement, n (%)**				
Family	300 (74)	236 (71)	64 (86)	<0.05
Alone	16 (4)	16 (5)	–	0.06
Alone with family or social support	71 (17)	64 (19)	7 (9)	<0.05
Not recorded	21 (5)	18 (5)	3 (4)	0.64
**Site of tumour, n (%)**				
Oral cavity	227 (56)	185 (55)	42 (57)	0.83
Oropharynx	47 (12)	45 (13)	2 (3)	<0.05
Nasopharynx	10 (2)	4 (1)	6 (8)	<0.05
Hypopharynx	13 (3)	11 (3)	2 (3)	0.79
Larynx	45 (11)	39 (12)	6 (8)	0.38
Unknown primary	11 (3)	10 (3)	1 (1)	0.43
Other^a^	59 (14)	44 (13)	15 (20)	0.12
**Stage of tumour, n (%)**				
0	3 (1)	3 (1)	–	0.41
1	138 (34)	114 (34)	24 (32)	0.78
2	74 (18)	60 (18)	14 (19)	0.85
3	61 (15)	48 (14)	13 (18)	0.49
4	111 (27)	92 (28)	19 (26)	0.74
Benign/Unknown/not recorded	21 (5)	17 (5)	4 (5)	0.91
**Intention of treatment, n (%)**				
Curative	382 (94)	310 (93)	72 (97)	0.15
Palliative	26 (6)	24 (7)	2 (3)	
**Modality of treatment, n (%)**				
Sx	168 (41)	127 (38)	41 (55)	<0.05
Sx with adjuvant or neoadjuvant	97 (24)	84 (25)	13 (18)	0.17
RT or CRT	132 (32)	113 (34)	19 (26)	0.17
CMT or IMT	11 (3)	10 (3)	1 (1)	0.43
**HPV (P-16) status, n (%)**				
Positive	62 (15)	60 (18)	2 (3)	<0.05
Negative	26 (6)	24 (7)	2 (3)	0.15
Awaiting or not recorded	320 (78)	250 (75)	70 (95)	<0.05
**BMI (Kg/m**^**2**^**), n (%)**				
<16	2 (0.5)	1 (0.3)	1 (1)	0.24
<18.5	15 (4)	12 (4)	3 (4)	0.85
18.5–24.9	155 (38)	129 (39)	26 (35)	0.58
25–29.9	150 (37)	124 (37)	26 (35)	0.75
>30	86 (21)	68 (20)	18 (24)	0.45
**Nutritional status**^b^**, n (%)**				
At risk of malnutrition	374 (92)	306 (92)	68 (92)	0.94
Malnourished	34 (8)	28 (8)	6 (8)	
**Anticipated need for enteral tube feeding during treatment, n (%)**				
Nasogastric tube	264 (65)	209 (63)	55 (74)	0.06
Gastrostomy tube	128 (31)	113 (34)	15 (20)	<0.05
Enteral tube feeding not anticipated	16 (4)	12 (4)	4 (5)	0.47

EPL = English primary language, NEPL = non-English primary language, HPV (P-16) = human papilloma virus type 16, Sx = surgery, CMT = chemotherapy, RT = radiotherapy, CRT = chemoradiotherapy, IMT = immunotherapy, BMI = Body Mass Index.

a Other refers to nose, sinus, thyroid, bone, skin, parapharyngeal space and saliva gland.

b Nutritional status defined by NICE guideline 2017 [CG 32].

**Fig. 1:**
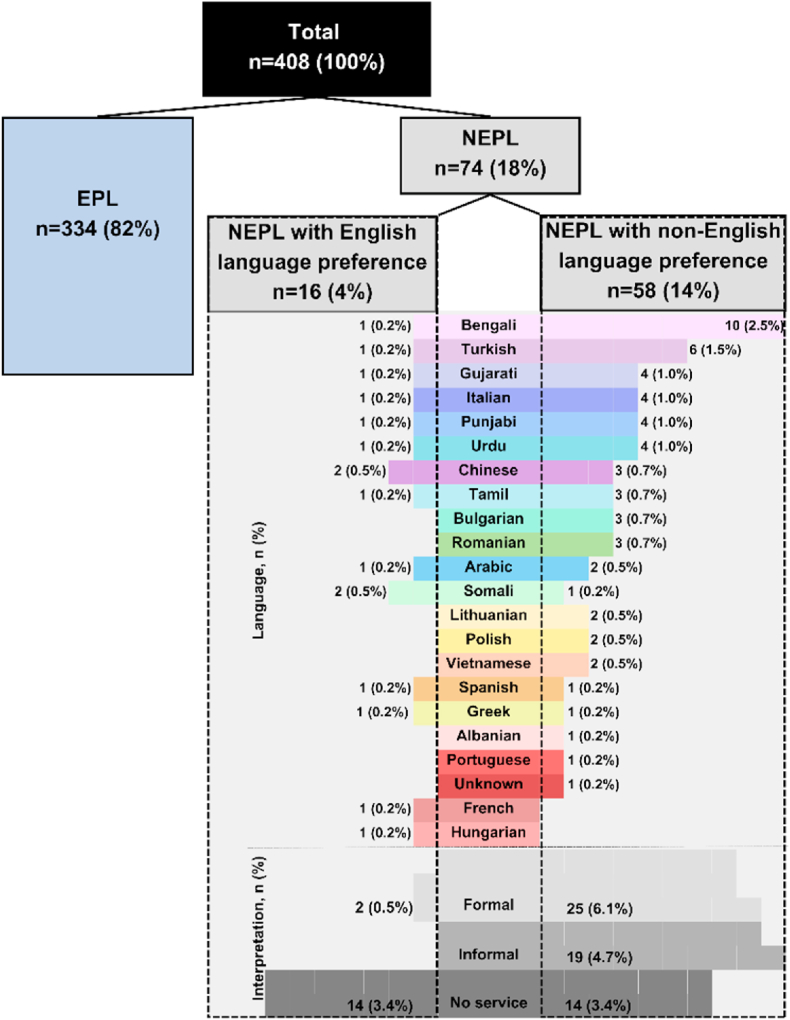
Count and proportion of patients across different languages, alongside the representation of interpretation services used Formal interpretation service operated by the hospital. Informal interpretation means using patients' relatives as translators. No service means not recorded utilisation of interpretation service. EPL = English primary language, NEPL = non-English primary language.

Primary language and interpretation services are depicted in Fig. 1. NEPL had diverse primary languages. Bengali (n = 11) and Turkish (n = 7) were most common. Among NEPL, non-English language preference patients outnumbered English language preference (n = 58, 78% vs. n = 16, 22%). In NEPL with non-English language preference (n = 58), formal interpretation service (n = 25, 43%) was most common, followed by informal service (n = 19, 33%) (using patients' relatives as translators), and no service (n = 14, 24%), respectively.

Table 2 illustrates languages and interpretation utilisation, with Turkish (n = 5, 9%) being most common in formal interpretation, Bengali (n = 6, 10%) in informal translators, and Gujarati (n = 4, 7%) in cases without interpretation. Information about written language requirements was limited as data was missing in 69% of cases.

**Table 2:** Spoken and written languages and uptake of interpretation services among patients with non-English language preference (n = 58).

	Total (n = 58)	Formal service^a^ (n = 25)	Informal service^b^ (n = 19)	No service^c^ (n = 14)
**Spoken language, n (%)**				
Arabic	2 (3.4)	2 (8.0)	–	–
Albanian	1 (1.7)	–	1 (5.3)	–
Bengali	10 (17.2)	3 (12.0)	6 (31.6)	1 (7.1)
Bulgarian	3 (5.2)	–	2 (10.5)	1 (7.1)
Chinese	3 (5.2)			
- Chinese Cantonese		1 (4.0)	–	–
- Chinese Mandarin		1 (4.0)	–	–
- Chinese Unidentified		–	–	1 (7.1)
Greek	1 (1.7)	1 (4.0)	–	–
Gujarati	4 (6.9)	–	–	4 (28.6)
Italian	4 (6.9)	1 (4.0)	2 (10.5)	1 (7.1)
Lithuanian	2 (3.4)	1 (4.0)	–	1 (7.1)
Polish	2 (3.4)	2 (8.0)	–	–
Portuguese	1 (1.7)	1 (4.0)	–	–
Punjabi	4 (6.9)	3 (12.0)	–	1 (7.1)
Romania	3 (5.2)	1 (4.0)	2 (10.5)	–
Somali	1 (1.7)	–	1 (5.3)	–
Spanish	1 (1.7)	1 (4.0)	–	–
Tamil	3 (5.2)	1 (4.0)	1 (5.3)	1 (7.1)
Turkish	6 (10.3)	5 (20.0)	–	1 (7.1)
Urdu	4 (6.9)	–	3 (15.8)	1 (7.1)
Vietnamese	2 (3.4)	1 (4.0)	–	1 (7.1)
Unknown	1 (1.7)	–	1 (5.3)	–
**Written language, n (%)**				
Abkhazian	1 (1.7)	–	–	1 (7.1)
Bengali	1 (1.7)	–	1 (5.3)	–
English	15 (25.9)	7 (28.0)	6 (31.6)	2 (14.3)
Romania	1 (1.7)	–	1 (5.3)	–
Not recorded	40 (69.0)	18 (72.0)	11 (57.9)	11 (78.6)

a Formal interpretation service operated by the hospital.

b Informal interpretation means using patients' relatives or friends as translators.

c No interpretation service means not recorded utilisation of interpretation service.

**Fig. 2:**
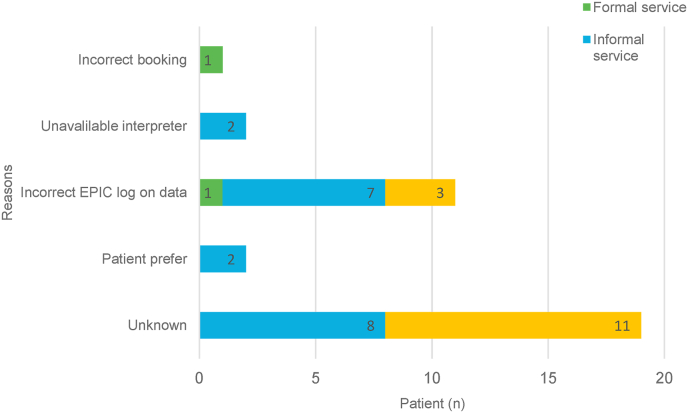
Reasons for inappropriate or inadequate use of interpretation services. Incorrect EPIC log on data’ refers to instances where incorrect log-on interpretation needs, no language data, or incorrect language data in the hospital record system led to misclassification, marking patients with English language preference as non-English language preference or vice versa.

Concerning the appropriate utilisation of interpretation service among NEPL, 39 (52.7%) appropriate arrangements were made. Among these, 25 patients used formal interpretation services appropriately, and 14 patients with English language preference did not require any interpretation service. Reasons for inappropriate interpretation service use are depicted in Fig. 2. Among 35 cases, the reasons were: not recorded (n = 19), language needs not documented in patient records (n = 11), patient preference (n = 2), no available interpreter (n = 2), and incorrect booking (n = 1). Different reasons appeared for each service. For patients receiving formal services, two patients with English language preference had interpreters booked but did not require them, one patient had an incorrect booking, and one was incorrectly marked as non-English language preference. In informal service, the reasons were: unknown (n = 8), language needs not documented in hospital records (n = 3), incorrectly marked as EPL (n = 2), incorrectly marked as English language preference (n = 2), expressed preference for family-assisted translators (n = 2) and interpreters not available for Bulgarian and Romanian languages (n = 2). In patients who did not receive interpretation services, n = 11 patients had unknown reasons and n = 3 did not have language needs documented in their records.

**Fig. 3:**
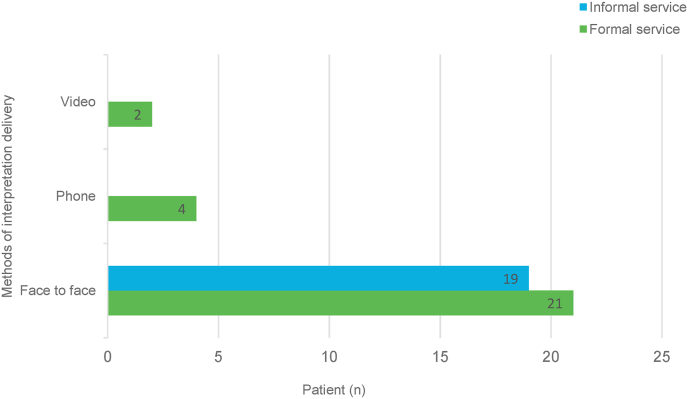
Methods of interpretation delivery. Formal interpretation: language line services operated by the hospital. Informal interpretation: using patients' relatives as translators. Nb. Formal service for n = 27 patients (includes the n = 2 with EPL that had formal interpretation booked incorrectly).

Interpretation delivery methods are depicted in Fig. 3. In formal services, face-to-face was most common (n = 21 78%), followed by telephone (n = 2, 15%), and video (n = 4, 7%), respectively. In informal services only face-to-face method was used (100%).

Out of the 444 visits, written information was provided in 438 visits. The most provided information was the leaflet on carbohydrate loading before surgery (n = 172), followed by the patient information booklet on decision making for a gastrostomy tube (n = 98), and swallowing exercises (n = 93), respectively.

## 4 Discussion

This audit aimed to identify and report interpretation needs of patients with HNC attending the joint dietitian/SLT pre-treatment clinic and adherence with NICE guidelines for providing interpretations services and reasons for any non-adherence. Of 74 patients with NEPL (non-English primary language), 58 (78%) had non-English language preference, while 16 (22%) had English language preference. In NEPL with non-English language preference patients (n = 58), formal interpretation (n = 25, 43%), informal interpretation (n = 19, 33%), and no service (n = 14, 24%) were used. This demonstrates that 100% adherence with NICE guidelines were not met in this service, as 24% of patients with non-English language preference did not have interpretation requirements met formally or informally. Reasons for non-adherence to formal interpretation included lack of language needs being documented in patient records, no interpreter available, patient preference, and ‘unknown’.

In this audit, surgical treatment was more common in NEPL (55%) than EPL (38%, p-value <0.05). This finding aligns with a study conducted in the United States where patients with limited English proficiency were more likely to receive primary surgery compared to primary non-surgical treatment after adjusting for clinical factors including tumour stage and primary site [15]. The factors influencing treatment decisions besides tumour stage might be cultural preferences, patient understanding of risks and benefits, and physician biases could contribute to different choices across populations. However, in this audit, multivariate analysis was not performed to determine whether there was an association between primary language and clinical factors.

This audit found that most patients were EPL (English primary language), aligning with the UK population predominantly being White British [16]. However, 18% were NEPL, and 14% were non-English language preference and faced language barriers. The high proportion of Bangladeshi patients is likely due to referrals to the centralised centre for surgery from East London, notably Tower Hamlets (34.7% Bangladeshi population) [17]. Notably, of the eleven Bangladeshi patients, the majority were non-English language preference (91%, n = 10). Data from the UK Census also indicates this area has higher deprivation levels. These findings, combined with “lower paying” occupational rates among NEPL patients compared to EPL, highlight potential socioeconomic differences. It is vital that these patients can access interpretation services to prevent inequalities in care.

Underutilisation of formal interpretation services due to lack of documentation of language needs in hospital records is consistent with findings from existing studies. A study conducted in the United States demonstrated successful achievement and maintenance in improving interpreter use by educating healthcare providers and patients with non-English language preference about the importance of utilising professional interpreters alongside improving health information technology (IT) [18]. Here, health IT enabled improved identification of language barriers, and access to various interpreting methods for requesting face-to-face interpreters by creating a specific section within the hospital record system to document interpreter usage.

Underutilisation of formal services due to lack of interpreter availability in specific languages indicates inequalities in care, and emphasises the need for equitable access to interpretation across diverse linguistic backgrounds.

Underutilisation due to patient preference, suggests the benefits of formal interpretation services are not well understood by patients, patients’ families, and carers. A study that interviewed patients with non-English language preference, revealed factors influencing the decision to use an interpreter included their English proficiency and the anticipated complexity of healthcare communication or information [19,20].

Lastly, underutilisation without recorded reasons might be attributed to various factors. In a previous study conducted in the inpatient setting, barriers such as time constraints have been reported [21]. Physicians often considered underusing professional interpreters as normal, even though patients with non-English language preference were not receiving equitable care [21]. In emergency settings, barriers have been reported to include limited knowledge amongst staff of requirements for interpretation, insufficient training, and lack of education on working with interpreters [22]. To address the unknown reasons behind the underutilisation of formal interpretation services, it is essential to implement a systematic approach for identification and resolution. This involves conducting comprehensive assessments, gathering feedback from healthcare providers and patients, and implementing targeted interventions aimed at improving the uptake of formal interpretation services [23].

### 4.1 Study limitations

This clinical audit had a relatively small sample size and is limited by missing data for a few variables such as reasons for not using formal interpretation services. Determination of language proficiency is not formally assessed routinely at the hospital. This meant that determining non-English language preference relied on healthcare staff recording language preferences, which lacks standardised English language competency screening validation [24]. Whilst this was a single-centre audit, findings are likely generalisable to a diverse HNC population. The nature of being a centralised London cancer centre is such that the centre received referrals from both the local inner-city population and across the south-east of England. Furthermore, it is a national centre for head and neck sarcoma subsite diseases in the UK, receiving referrals from across the country.

### 4.2 Implications for practice

This audit raises important implications for practice to improve equality, diversity, and inclusion of patient care locally and perhaps more widely. Based on the audit, we suggest the following be considered:

Firstly, to increase awareness amongst healthcare providers and patients about using formal interpretation services and avoiding informal interpreters to avoid miscommunication, misunderstanding and to uphold ethical principles and validity of consent [25]. This could be accomplished by enhancing cultural competence [26], training on addressing language barriers and working with interpreters [22] and encouraging documentation of language and interpretation requirements in medical records. By avoiding the use of the default recording system, inaccuracies in information can be prevented, particularly the primary language and the requirement for an interpreter.

Secondly, to enhance health IT systems to facilitate information sharing between hospitals, including language barriers and interpretation requirements of patients [18]. This may depend on staff engagement with training for this and the provision of resources to facilitate change.

Thirdly, to ensure commissioning of equitable interpretation services tailored to the local population [25,27]. In our centre, this could include expanding the pool of professional interpreters available to ensure all patients have access to services when required, as this audit found that Bulgarian and Romanian interpreters were not available. Although this may not be feasible in practice for all languages, some could be prioritised based on the findings of this audit [27]. In addition, using artificial intelligence such as multilingual chatbots which use natural language processing technology to provide patients with live support in their preferred language [25].

Lastly, to make written information more routinely available in various languages. In this cohort, this could commence with translation of the most frequently used resources (pre-surgery carbohydrate loading leaflets, gastrostomy tube and swallowing exercises patient information booklets, into the most common languages (Bengali and Turkish). In circumstances where written information translation may not be appropriate, e.g. when it is known that spoken and written language literacy are discordant, the use of video link with oral transmission may be more effective [25].

### 4.3 Future work

Further research should explore adequate provision of interpretation services and patients' and staffs’ satisfaction of using formal interpretation services. This can help identify barriers and facilitators to optimal use of these services to promote patient and staff engagement. A national, multicentre audit or the inclusion of a more extensive and diverse patient population could expand on the findings from this audit.

## 5 Conclusion

This audit of 408 patients revealed that over a 2-year period, 74 patients (18%) were NEPL, with 58 patients (14%) having non-English language preference. Among those with non-English language preference, 43% used professional interpreters, 33% used informal translators, and 24% did not use interpreters. Reasons for underutilisation of formal interpretation were attributed to the lack of documentation of language needs, interpreter availability, and patient preference. This audit indicates the need to raise awareness about formal interpretation services and provide necessary language support in keeping with recommended guidelines that promote equality, diversity, and inclusion.

## Funding statement

Florence Cook was funded through a University College London Hospital Charity Chief Nurse Research Fellowship. Roganie Govender is funded by the National Institute of Health and Care Research (NIHR Clinical Lecturer fellowship NIHR300427). The views expressed in this publication are those of the author(s) and not necessarily those of the NIHR, NHS or UK Department of Health and Social Care.

## CRediT authorship contribution statement

**Maesaya Chartkul:** Conceptualization, Data curation, Formal analysis, Investigation, Methodology, Project administration, Resources, Software, Validation, Visualization, Writing – original draft, Writing – review & editing. **Florence Cook:** Conceptualization, Methodology, Supervision, Writing – review & editing. **Roganie Govender:** Conceptualization, Methodology, Supervision, Writing – review & editing.

## Declaration of competing interest

The authors declare that they have no known competing financial interests or personal relationships that could have appeared to influence the work reported in this paper.
